# Comparative Analysis of Normal versus Fetal Growth Restriction in Pregnancy: The Significance of Maternal Body Mass Index, Nutritional Status, Anemia, and Ultrasonography Screening

**DOI:** 10.1155/2013/671954

**Published:** 2013-12-29

**Authors:** Laxmichaya D. Sawant, Shirin Venkat

**Affiliations:** Department of Obstetrics and Gynecology, Grant Medical Foundation, Ruby Hall Clinic, Pune, Maharashtra 411001, India

## Abstract

Fetal growth restriction or intrauterine growth restriction is one of the leading causes of perinatal mortality and morbidity in newborns. Fetal growth restriction is a complex multifactorial condition resulting from several fetal and maternal disorders. The objective of this study was twofold: first to examine the correlation between maternal parameters such as body mass index (BMI), nutritional status, anemia, and placental weight and diameter, and their effects on fetal growth and then to evaluate the effect of early screening by ultrasonography (USG) on the outcome of growth restricted pregnancies. In this study, 53 cases of fetal growth restriction were compared to 53 normal fetuses delivered in consecutive sequence. Growth restricted fetuses were delivered earlier in gestation, when compared with normal growth fetuses. Maternal anemia and malnutrition have significant association with the fetal growth restriction. Maternal anthropometry, such as low BMI, had effects on placental diameter and weight, which, in turn, adversely affected fetal weight. Thus, early USG screening along with robust screening for maternal BMI, nutritional status, and anemia can assist the obstetric team in providing early diagnosis, prompt intervention, and better outcome in pregnancy with fetal growth restriction.

## 1. Introduction

In this century, fetal growth restriction continues to be a significant perinatal problem [1]. A growth restricted fetus is one with an estimated fetal weight of less than the tenth percentile for that gestational age [2]. The prevalence of growth restricted fetuses is known to be about 10% [3]. The incidence of fetal growth restriction varies depending upon the population residing in the developing and developed countries with a incidence rate of 6–30% to 2–5% in these countries, respectively [4, 5]. The highest rate of prevalence of fetal growth restriction is found in Asia, particularly in Southeast Asia, followed by Africa and Latin America [6, 7]. These statistics make the fetal growth restriction during pregnancy a major public health concern throughout the world, especially in the developing countries with a huge population base, a lack of good and affordable health care infrastructure, and a low patient-physician ratio.

Fetal growth restriction or intrauterine growth restriction (IUGR) cannot be termed into a specific disease entity per se, but it is rather a complex multi-factorial condition. It is manifested as a result of several fetal and maternal disorders [2]. The factors affecting fetal growth restriction are the nature of the etiological agents and the duration of gestation [8]. These factors can be classified into maternal, fetal, placental, and environmental factors. The maternal factors consist of preeclampsia, diabetes mellitus, and heart diseases. The fetal factors include aneuploidy, chromosomal abnormalities, and multiple gestation. The placental factors comprise placenta previa, placenta accreta, abruptio placentae, and finally the environmental factors, such as smoking, drugs, maternal malnutrition, illiteracy and low socio-economic status are involved in fetal growth restriction [3, 6, 9, 10].

The fetal growth restriction makes the fetus more prone to perinatal morbidity and mortality due to the failure of a fetus to attain its complete growth potential [2, 3]. It also increases its risk for long term consequences, such as coronary heart disease, type-2 diabetes mellitus, hypertension, and metabolic syndrome [3, 11, 12]. Therefore, having knowledge of predisposing extrinsic factors may help in early diagnosis, prompt intervention and better management, which can ultimately lead to good obstetric care during fetal growth restriction. In this study, our objective was twofold: first to examine the effect of maternal parameters such as body mass Index (BMI), nutritional status, anemia on placental weight and diameter, followed by its effect on the growth of the fetuses. And, secondly, to see the effect of early screening by ultrasonography (USG) on the outcome of growth restricted pregnancies.

## 2. Materials and Methods

The present study was carried out in the Department of Obstetrics and Gynecology with proper approval from the Director of Academics and Research (Education) office in Ruby Hall Clinic, Pune, India. The period of study was from February 2007 to February 2008. The total number of deliveries reported in this period was 820, out of which 53 cases were of growth restricted fetuses. For this study, 53 cases of normal fetuses were selected randomly and compared with 53 cases of growth restricted fetuses. The criteria considered for the growth restricted pregnancy were gestational age of more than 26 weeks and all fetuses with a weight of less than tenth percentile for that gestation.

Mothers were screened in the antenatal period for fetal growth restriction, and those patients with complications like gestational hypertension, anemia, and heart disease were admitted into the ward. They were given appropriate treatment after doing relevant investigations and were followed until delivery. Out of 106 cases studied, patients with fetal growth restriction were admitted in antenatal period. There were no incidences of twin birth or still birth during this period. The remaining cases were admitted to labor room directly. All the above cases were registered; during their antenatal visits, mothers were asked about their dietary history, such as how many times do you have food in a day? and how many servings of chapattis (Indian bread), dal (pulses), vegetables, beans, rice, meat, fish, and eggs do you have per day including breakfast, lunch, dinner, and daily snacks? Then, accordingly, carbohydrate, protein, and fat contents were calculated.

After delivery, the neonatologist examined fetuses; fetuses were weighed and their gestational ages were estimated. On delivery the placenta was examined immediately in fresh state. The excess blood was washed out under running water. The placenta was then weighed and its diameter was calculated using maximum and minimum length, that is the distance calculated from the cord to the periphery of the placental on each side.

### 2.1. Statistical Analysis

The statistical analysis was performed utilizing SPSS software version 11 (chi-square test, *Z*-test) and the significance level of *P* value <0.05 was accepted as statistically significant.

## 3. Results

The total number of deliveries that took place during the period of study was 820, out of which, the infants with fetal growth restriction were 53, giving an incidence of 6.4% for fetal growth restriction in our study. All the cases collected were registered cases in Ruby Hall Clinic and Urban Health Centre of Ruby Hall Clinic, Pune, India. The comparison of gestational age by USG in our study groups was found to be statistically significant. These numbers indicate that growth restricted fetuses were highest at 35 weeks in our study group (Table 1; *P* < 0.0001). The comparison of hemoglobin in our study groups showed statistically significant differences. This indicates that a decrease in maternal hemoglobin could play a role in restricting growth in fetuses (Table 2; *P* < 0.05). Similarly, comparison of nutritional status showed a statistically significant difference between mothers of growth restricted and normal fetuses. This result further confirmed the role of malnutrition in fetal growth restriction (Table 3; *P* < 0.0001). Moreover, we have also statistically compared BMI in our study groups. We found that mothers with fetal growth restriction have low BMI compared to mothers with normal fetuses (Table 4; *P* < 0.0001).

**Figure 1:**
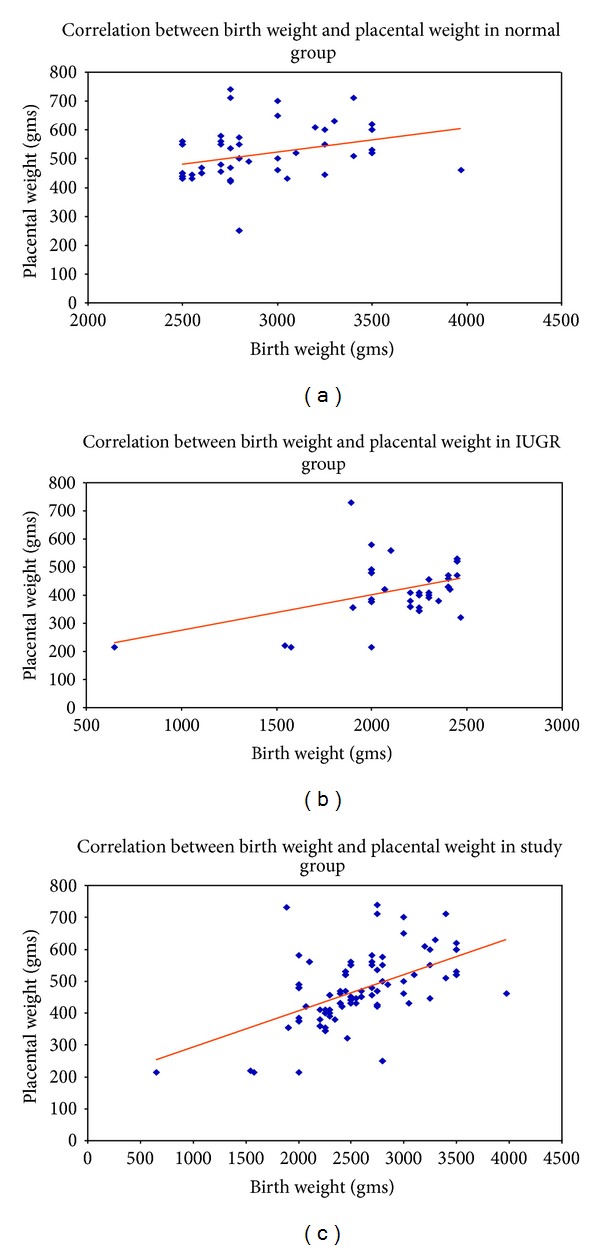
(a) The scatter diagram showing the correlation between birth weight and placental weight in the normal group. (b) The scatter diagram showing the correlation between birth weight and placental weight in the group with fetal growth restriction/intrauterine growth restriction (IUGR). (c) The scatter diagram showing the correlation between birth weight and placental weight in the entire study group combining normal fetuses and group with fetal growth restriction/IUGR.

On comparing the placental weight in the study groups, we found that fetal weight decreases as placental weight decreases (Table 5; *P* < 0.0001). Also, the difference in the ratio between fetoplacental weight in the normal versus growth restricted fetuses is statistically significant (Table 6; *P* < 0.05). Furthermore, we also showed a statistically significant correlation between birth weight and placental weight in the fetuses with normal weight, growth restricted fetuses (IUGR), and the entire study population by combining both normal and fetal growth restricted study groups (Table 7; *P* < 0.0001). The results showing correlation between birth weight and placental weight in the fetuses are also demonstrated in a scatter diagram format for each separate study group (Figures 1(a), 1(b), and 1(c)). We have also compared the placental diameter in the study groups, which demonstrated that the decrease in the placental diameter was less in growth restricted fetus compared to normal fetus (Table 8; *P* < 0.01).

## 4. Discussion

The incidence of growth restricted fetuses in our study was 6.4%. We also demonstrated that utilizing USG screening early in the pregnancy helps in early diagnosis, better management, and better outcome in growth restricted fetuses. In our study, none of the normal fetuses showed abnormal placenta. Placental diameter and weight were significantly reduced in growth restricted deliveries. Maternal anthropometry, anemia, and nutritional status had an effect on fetal birth weight which is likely to be mediated by its effect on placental volume. Thus, this study demonstrates that the study of placental morphology in fetal growth restriction is an important factor for good fetal outcome. Furthermore, this study has also demonstrated that by keeping maternal factors such as BMI, anemia, and nutritional status in check, we can significantly improve placental growth, which, in turn, will affect the weight of the fetus resulting in a favorable obstetric outcome.

The incidence of fetal growth restriction in pregnancies of 6.4% in the present study was less compared to that of 9.7% and 12% as stated in other studies [5, 13]. This could be due to appropriate and intensive antenatal care given to the patients in our study. We have noticed that when a comparison of gestational age was done by USG, the mean gestational age for normal fetus was 38.22 weeks, while that of growth restricted fetuses was 35.80 weeks. Researchers have shown earlier that since USG was used as a diagnostic tool to monitor fetal growth restriction, that could have led to early intervention and early birth of growth restricted fetuses. This was reflected as an increase in the number of cases of fetal growth restriction in premature infants [14].

The hemoglobin levels in our patients from the study population showed a mean of 10.96% hemoglobin levels in fetal growth restricted pregnancies compared to mean of 11.48% in normal pregnancies. Similarly, other studies have also shown the presence of maternal anemia in their fetal growth restricted cases in the range of 8.5% and 10% [5, 15]. The study of the nutritional status in our study groups showed a statistically significant difference between fetal growth restriction and normal pregnancies. This proved that maternal nutrition plays a vital role in the cases of fetal growth restriction and it can be inferred that if maternal nutrition is hampered, then growth of fetus will be jeopardized. When we compared maternal BMI in our study groups, we found that the mean BMI in mothers having normal fetuses was 26.16, whereas in mothers who had fetal growth restricted infants, the BMI was 22.58. This finding was statistically very significant and it reaffirmed that during pregnancy, the maternal BMI has a critical role in intrauterine fetal growth and development. It has been demonstrated that the effects of maternal anthropometry on birth weight are likely mediated through the effects of maternal anthropometry on placental volume [16].

In our study, we found a strong correlation between fetal weight and placental diameter as well as placental weight. We observed that as fetal weight decreases, the placental diameter along with placental weight decreases. However, in most instances, no obvious maternal or fetal cause could be found, yet the infants had a profound reduction in weight. In such cases, it has been implicated that the fault lies in the placenta. One study has stated that placental insufficiency leads to a syndrome of fetal compromise with fetal weight deficit [17]. In general terms, the placental weight is related to fetal birth weight. Association between fetal and placental weight was recognized as early as the 19th century. As gestational age increases, placental weight also increases. However, it is not clear if the placenta does so by increasing its weight and thickness. Some studies have demonstrated the relationship between fetal weight and placental growth. The results have shown that when the fetus is small, the placenta being a fetal organ shows diminished growth along with the reduction in the placental weight [18, 19].

Researchers who studied placentas in growth restricted fetuses also stated that the majority of placentas showed fibrin deposits, infarcts, and overgrowth of trophoblastic tissue. They also noticed nonspecific inflammation of placental villi with loss of vascularity with the apparent site of injury being placental syncytiotrophoblast layer [17]. Another research group found that placentas of growth restricted fetuses were more frequently infiltrated with leukocytes [20]. In such placentas, the blood vessels were seen occluded due to deposits, resulting in ischemic damage to the placenta. Similar placental infarcts were commonly found in women with hypertension; however it can be even found in normotensive women [21, 22]. Researchers have demonstrated that, with the increase in the severity of toxemia in pregnancy, all the placental changes are exaggerated. Moreover, it has been documented that in fetal growth restriction pregnancy, which is complicated by maternal gestational hypertension, there is acceleration in the process of the placental aging with subsequent reduction in its functional capacity, resulting in a greater degree of placental inflammation [21].

## 5. Conclusion, Significance, and Recommendation

In summary, the results of this study demonstrated that early screening of pregnant women for BMI, maternal nutrition, anemia, and gestational age using USG can effectively decrease fetal growth restriction. However, the results of this research study do not rule out the cumulative effect of the low maternal nutritional status, low BMI, and anemia on fetal growth restriction. Since fetal growth restriction is one of the preventable obstetric complications seen in developing countries especially in Southeast Asia, Africa, and Latin America, more efforts need to be done to curtail this perinatal issue by carrying out more research highlighting the probable factors affecting growth of the fetus. Therefore, the strength of this study is that it reaffirms the implication of USG screening in the management of fetal growth restriction in terms of achieving early detection and prompt intervention, thereby reducing perinatal fetal mortality and morbidity. The present research study also has some weakness in terms of not having larger sample size. Also, the study doesnot provide in-depth nutritional analysis of the subjects in the research groups, which, in turn, can provide better comparison between each specific nutritional component such as protein, fat, and carbohydrate, along with micronutrients such as vitamins, iron, and calcium, among the subjects with fetal growth restriction. Since the current study cannot differentiate between the confounding effect of the variables, the future research study should be designed with multivariate regression modeling to evaluate the effect of each of the above variables on fetal growth and thus minimizing their confounding effect on fetal growth restriction.

Moreover, looking through a public health perspective, this study also highlights the importance of maternal BMI, nutrition, and anemia on fetal growth and development. However, in the future, more clinical and population based studies should be carried out on the global health platform looking in to epidemiological and socio-behavioral aspects of maternal nutrition with respect to cultural, racial, and ethnic variations in demography affecting dynamics of fetal life during pregnancy. We have also propose that more studies performing qualitative and quantitative assessment of the placenta in relation to fetal outcome are required to better understand the placental changes in presence of different variables affecting fetal growth and development during gestation period.

Thus, the study recommends that along with maternal education about pregnancy and related prenatal, antenatal, and postnatal child health care, there should be an effective public health intervention in improving maternal nutrition. Furthermore, the medical facilities, such as rural hospitals and primary health care clinics, should be equipped with cost-effective screening tools such as USG machines. Moreover, clinical staff such as medical doctors, nurses, and midwives working at primary health care facilities should be properly trained in acquiring additional skills in diagnosing fetal growth restriction through USG. These approaches at primary, secondary, and tertiary prevention levels, respectively, will collectively provide a synergistic effect in decreasing prevalence and incidence of fetal growth restriction in pregnancies. In conclusion, the study strongly advocates a multidirectional approach in order to develop preventive and therapeutic strategies against fetal growth restriction in pregnancy, thereby impacting maternal and child health globally.

## Figures and Tables

**Table 1:** The comparison of gestational age (GA) by ultrasonography (USG) in study groups.

Gestational age (GA)	Normal fetus	Growth restricted fetus	*Z* value	*P* value
	Mean ± SD (*n* = 53)	Mean ± SD (*n* = 53)		
GA (wks)	38.22 ± 1.31	35.80 ± 2.95	5.46	<0.0001

**Table 2:** The comparison of hemoglobin (Hb) in study groups.

Hemoglobin (Hb)	Normal fetus	Growth restricted fetus	*Z* value	*P* value
	Mean ± SD (*n* = 53)	Mean ± SD (*n* = 53)		
Hb. (gms %)	11.48 ± 1.14	10.96 ± 1.32	2.17	<0.05

**Table 3:** The comparison of maternal nutritional status in study groups.

Nutritional components	Normal fetus	Growth restricted fetus	*Z* value	*P* value
	Mean ± SD (*n* = 53)	Mean ± SD (*n* = 53)		
Carbohydrate (gms)	279.26 ± 3.56	266.30 ± 12.41	7.31	<0.0001
Protein (gms)	138.59 ± 5.87	126.76 ± 5.49	10.72	<0.0001
Fats (gms)	37.87 ± 5.08	29.43 ± 3.41	10.05	<0.0001

**Table 4:** The comparison of body mass index (BMI) in study groups.

Body mass index (BMI)	Normal fetus	Growth restricted fetus	*Z* value	*P* value
	Mean ± SD (*n* = 53)	Mean ± SD (*n* = 53)		
BMI	26.16 ± 1.89	22.58 ± 2.09	9.24	<0.0001

**Table 5:** The comparison of placental weight in study groups.

Placental weight	Normal fetus	Growth restricted fetus	*Z* value	*P* value
	Mean ± SD (*n* = 53)	Mean ± SD (*n* = 53)		
Placental weight (gms)	512.74 ± 98.18	420.85 ± 94.79	4.90	<0.0001

**Table 6:** The comparison of fetoplacental weight ratio in study groups.

Fetoplacental weight (F-P) ratio	Normal fetus	IUGR fetus	*Z* value	*P* value
	Mean ± SD (*n* = 53)	Mean ± SD (*n* = 53)		
F-P weight ratio	5.87 ± 1.38	5.31 ± 1.13	2.26	<0.05

**Table 7:** The correlation between birth weight and placental weight in the study groups.

Study group	Relation between	Correlation (*r*)	*P* Value
Normal fetus	Birth weight and placental weight (Gms)	0.31	<0.0001
Growth restricted fetus (IUGR)	Birth weight and placental weight (Gms)	0.40	<0.0001
All fetuses	Birth weight and placental weight (Gms)	0.54	<0.0001

**Table 8:** The comparison of placental diameter in study groups.

Placental diameter (Cms)	Normal fetus	Growth restricted fetus	*Z* value	*P* value
	Mean ± SD (*n* = 53)	Mean ± SD (*n* = 53)		
Maximum	17.11 ± 2.77	15.45 ± 3.72	2.61	<0.01
Minimum	13.51 ± 2.31	11.94 ± 1.44	3	<0.001
